# Supplementary material for: A DNA Finite-State Machine Based on the Programmable Allosteric Strategy of DNAzyme
Source: Int J Mol Sci. 2023 Feb 10;24(4):3588. doi: 10.3390/ijms24043588 (PMC9963683; doi:10.3390/ijms24043588)
Supplement: Supplementary file 1 [file ijms-24-03588-s001.zip › ijms-2131885-SI.pdf]

Supplementary

Contents

S1. Parameter optimization of DNAzyme cleaves substrate..................................2
S2. Reverse inhibition reaction of the state machine 4
S3. Design of state machine reversible transition..6
S4. Verification of state machine's controllability and extensibility.......................7
S5. DNA sequence 9

S1. Parameter optimization of DNAzyme cleaves substrate

To further optimize the reaction rate of the programmable allosteric strategy of DNAzyme, we performed a parametric analysis of the cleavage rate for the substrate RNA1. Firstly, we tested the effect of different base numbers in domain a on the cleavage rate of the substrate, as shown in Figure S1B. The results show that when the domain a was 15 nt, the effect of the cleavage reaction is optimum. Therefore, we finally chose the loop length of 15nt as subsequent the experimental parameter.

Then, we tested the effect of different base numbers in domain b on the cleavage rate of the substrate, as shown in Figure S1C. The results show that when domain b is 7 nt, the effect of the cleavage reaction is optimum. Therefore, we finally chose the stem length of 7 nt as the subsequent experimental parameter.

Finally, we also explored the effect of the base numbers of the domain d on the cleavage rate of substrate, as shown in Figure S1D. It is observed that DNAzyme activity gradually improves with the increase of base number, and the cleaved effect is preferred when the base number is 3 nt or 4 nt. We chose 3 nt as a subsequent experimental parameter because the sequence should avoid redundancy in the complex.

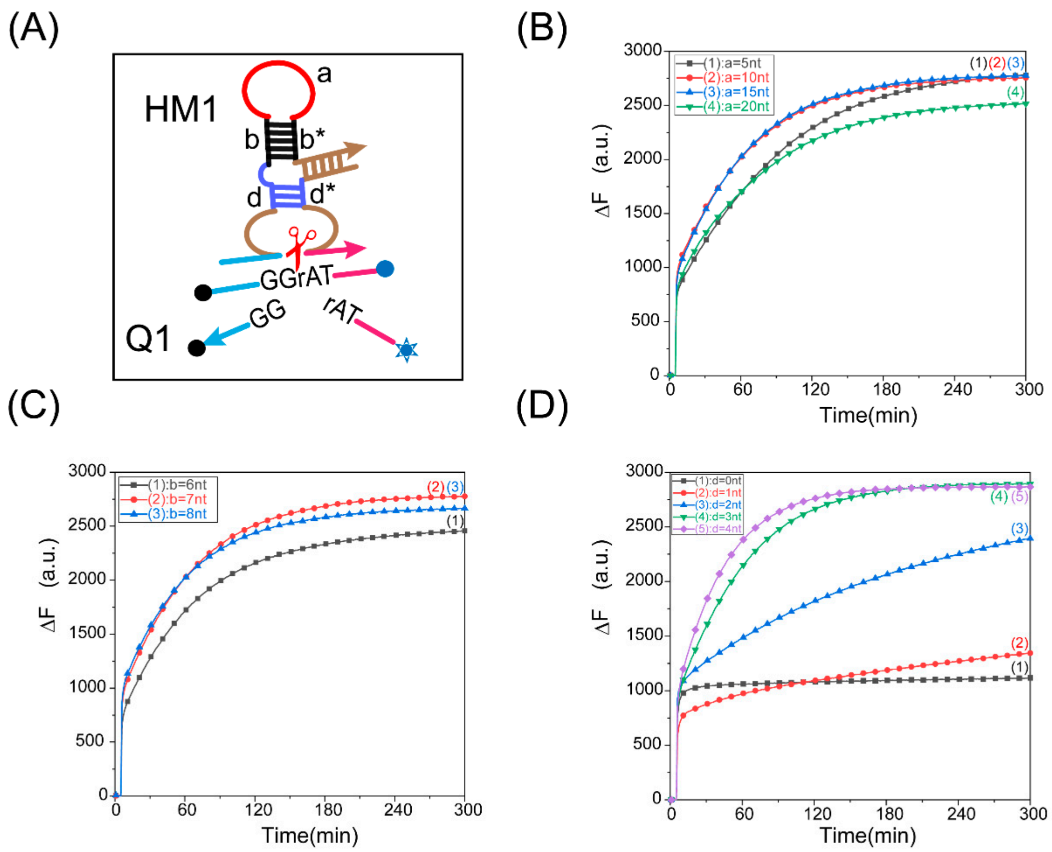

Figure S1. The parameter optimization of DNAzyme cleaved substrate. (A) Schematic diagram of DNAzyme cleaved substrate. (B) Fluorescence experiments with different numbers of bases in the domain a of DNAzyme. (C) Fluorescence experiments with different numbers of bases in the domain b of DNAzyme. (D) Fluorescence experiments with different numbers of bases in the domain d of DNAzyme.

S2. Reverse inhibition reaction of the State machine

In order to explore the function of reversible conversion of the state machine, we first verified the reverse inhibition process of the programmable allosteric strategy of DNAzyme, as shown in Figure S2A. The strand T1 hybridized with the loop portion of the DNA hairpin with the addition of T1, and the rigid linear double helix structure forced DNAzyme's conserved domain to separate, resulting in a complex Sub1 formed that does not have the ability to cleave the substrate RNA1.

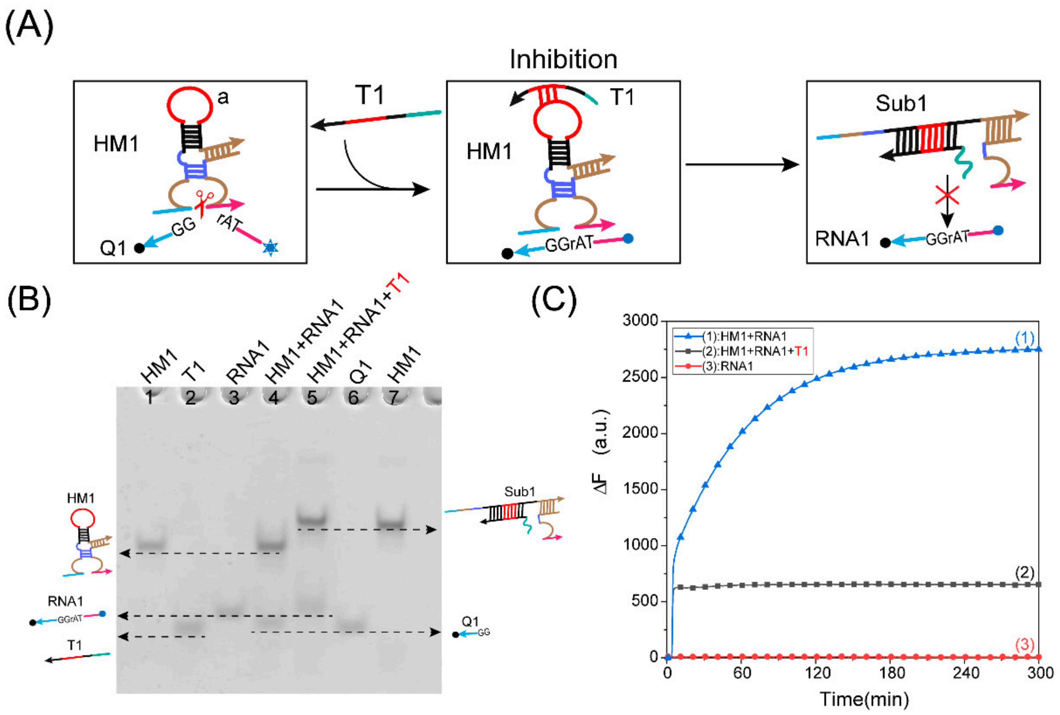

Figure S2. (A) The diagram of state machine reverse transition. (B) PAGE analysis of state machine reverse transitions. The strands and complexes involved are marked above the gel. (C) Fluorescence analysis of the state machine reverse transition. Curve (1), The initial solution contained the DNAzyme HM1 and the substrate RNA1. Curve (2), the input strand T1 was added to the solution containing the DNAzyme HM1 and the substrate RNA1. [HM1]:[T1]:[RNA1] = 1:1:1 = 0.1 μM. Curve (3), only substrate strand RNA1 existed in the solution.

Then, we performed a polyacrylamide gel electrophoresis experiment to verify the inhibition process of the state machine as shown in Figure S2B. In lane 4, HM1 cleaved substrate RNA1 without adding inhibitor T1, and it can be observed that substrate RNA1 disappeared and formed the cut product Q1. In lane 5, when the inhibitor T1 was added, DNAzyme disappeared and formed the three-stranded complexes Sub1 and the uncleaved substrate RNA1. In addition, we also performed fluorescence analysis as shown in Figure S2C. Curve 3 shows that only the substrate RNA1 was present in the solution; Curve 2 shows that the inhibitor T1 was added to the HM1 and RNA1 mixed solution initially, and the fluorescence curve did not rise, indicating that the inhibitory effect was excellent. This is in stark contrast to curve 1 which contains a mixed solution of HM1 and RNA1 and produces a significantly increased fluorescence signal.

S3. Design of state machine reversible transition

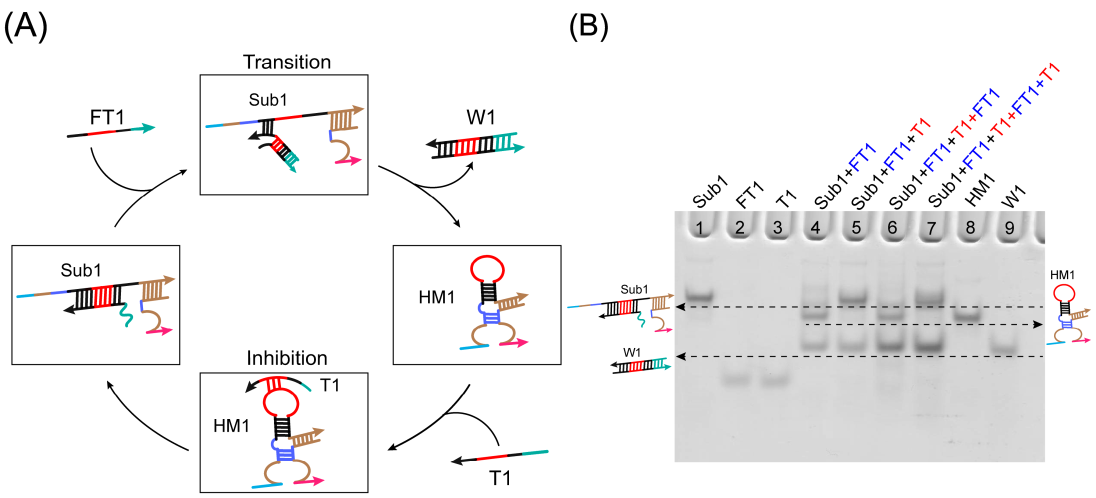

Figure S3. (A) Abstract diagram of the reversible transition of the state machine. (B) PAGE analysis of the reversible transition of the state machine.

To verify the dynamic regulation and reversible transition capabilities of the finite state machine, we analyzed the results of the polyacrylamide gel electrophoresis experiments. To make gel electrophoresis gel lanes more obvious, we only showed reversible transformations of structures. As shown in Figure S2B, when the input strand FT1 was added, it bound to the inhibitor T1 to form a double-stranded structure W1, and the remaining structure spontaneously formed an active DNAzyme. Therefore, in lane 4, we can observe that substrate Sub1 disappeared, and the double-stranded structure W1 and DNAzyme structure HM1 were formed. The strand T1 hybridized with the loop portion of the DNA hairpin with the addition of T1, and the rigid linear double helix structure forced the DNAzyme's conserved domain to separate. Therefore, in lane 5, we can observe that substrate HM1 disappeared, and the complex Sub1 was formed again. Therefore, we executed the sequential response of the temporal signal by alternately adding the input strand FT1 and the inhibitor T1 to realize the function of dynamic response and reversible switching of the finite-state machine.

S4. Verification of state machine's controllability and extensibility

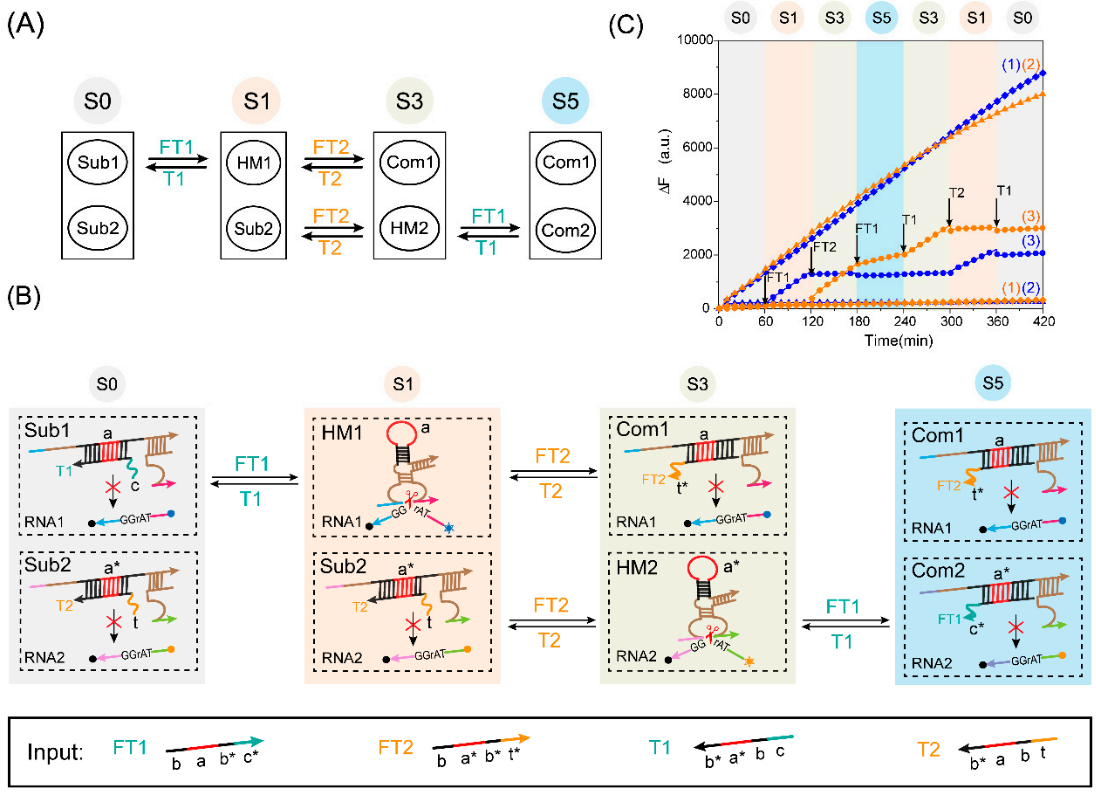

Figure S4. (A) Abstract diagram of four state linear loop transitions of the state machine. (B) Schematic diagram of four state linear loop transitions of the state machine. The blue circle represents FAM fluorescence and the orange circle represents ROX fluorescence. (C) Fluorescence analysis of the state cycle transitions.

To illustrate its controllability and expansibility, a biochemical framework grounded in this strategy is presented to determine four state linear loop transitions of the state machine. Our results highlight the potential for DNA state machines to act as signal controllers and event triggers to endow molecular systems with the capability of controlling and detecting events and signals. In Figure S4A, the abstract diagram schematically depicts the four states of the state machine. The state machine maintains a state represented by the numbered circles {S0, S1, S3, S5}, and each state contains two morphological structures. The arrows in the diagram represent starting from the current state and ending in the next state. The label on the arrow gives the corresponding input signal. The transition between each state is triggered by adding input signals.

Figure S4B shows in detail the morphological structures contained in each state and the transition process. When the state machine was in the S0 state, it contained complex Sub1 and complex Sub2. The difference between complex Sub1 and complex Sub2 was that DNA hairpin base sequences with molecular conformational changes were complementarily paired (labeled with a and a* in the figure). The strand FT1 underwent a strand displacement reaction from the toehold domain of the complex Sub1 when the strand FT1 was added, thus transitioning to the S1 state containing HM1 and Sub2. Notably, the de-sign highlight of this module is that it provides two functional domains for the input strand to perform dual roles, where the input

strand can not only activate DNAzyme activity by the strand displacement reaction from the toehold domain but also inhibit another DNAzyme activity by a hybridization chain reaction from the loop region of the DNA hairpin. Then, when FT2 was added, the strand FT2 could perform a hybridization chain reaction from its domain a* with the domain a of the HM1 structure to inhibit DNAzyme HM1 activity, and also perform a strand displacement reaction from the toehold domain of complex Sub2 to activate DNAzyme HM2. This resulted in a transition from the S1 state to the S3 state containing Com1 and HM2. Then, when FT1 was added, the strand FT1 could perform a hybridization chain reaction from its domain a with the domain a* of the HM2 structure to inhibit DNAzyme HM2 activity. This resulted in a transition from the S3 state to the S5 state containing Com1 and Com2. HM2 was activated again with the addition of T1, which made the state transform back to the S3 state. HM1 was activated again with the addition of T2, which made the state transform back to the S1 state. Finally, the state machine is returned to its original S0 state by adding T1.

To verify the feasibility of the state machine with the four states linear loop transition functions, we performed a real-time fluorescence experiment to show that the state machine can respond dynamically according to different temporal inputs. As shown in Figure S4C, we conducted positive control experiments at the same time. Curve 1 represents that only input strand FT1 was added to the complex Sub1, complex Sub2, substrate RNA1, and substrate RNA2 mixed solution. The FAM fluorescence shows an upward trend and no change in ROX fluorescence is observed, indicating that only HM1 was activated. Curve 2 represents that only the input strand FT2 was added to the complex Sub1, complex Sub2, substrate RNA1, and substrate RNA2 mixed solution. The ROX fluorescence shows an upward trend, and the FAM fluorescence signal does not increase, indicating that only HM2 was activated. Curve 3 starts with state S0, and neither the FAM fluorescence signal nor the ROX fluorescence signal changes. The DNAzyme HM1 was activated with the addition of FT1, but Sub2 remained unchanged. At this time, the FAM fluorescence rose, and the ROX fluorescence remained level, and the state machine was in the S1 state. Then, we added input FT2 after 60 min of reaction Due to FT2 having a dual role, FT2 could perform the strand displacement reaction from the toehold domain with Sub2 to activate DNAzyme HM2 activity, resulting in an upward trend in the ROX fluorescence. The reaction speed quickly reached the same level as the positive control. FT2 could also perform a hybridization chain reaction from the loop portion of the HM1 that caused HM1 to become Com1, and at this time, the FAM fluorescence tended to be horizontal and did not rise, indicating that the activity of DNAzyme HM1 was inhibited, and the state was S3. State 3 transitioned to state 5 after continuing to add FT1, and both the FAM fluorescence and the ROX fluorescence remained level. Immediately after strand T1 was added, HM2 was activated again, and the state transitioned from S5 to S3 again. Then, after strand T2 with the same dual effect as FT2 was added, HM1 was activated, HM2 was inhibited and the state transitioned from S3 to S1 again. Finally, the state returned to the initial state S0 after the addition of strand T1, at which point neither fluorescence was rising.

S5. DNA sequence

In order to make gel electrophoresis gel lanes more obvious, all the DNA sequences

in this experiment were designed by NUPACK. At the same time, in order to make gel electrophoresis lanes more obvious, multi-thymine (T) was employed. DNA sequences are shown in Table S1

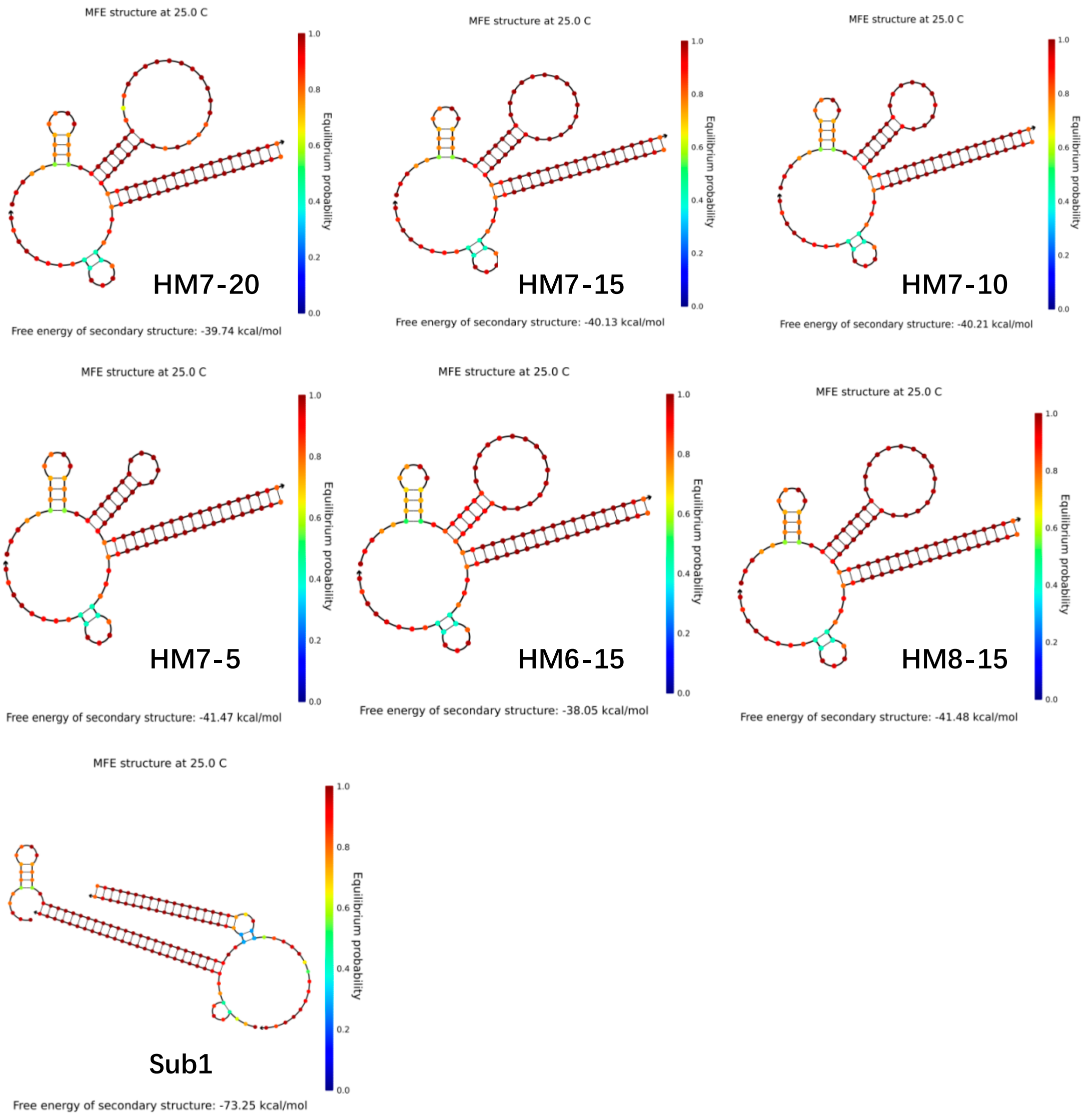

Table S1 DNA sequences in this study

Name	Sequences (from 5′ to 3′)	Length (n.t.)
**D1**	TGACGACAGCGATCTGTTGCCTGACTCTCACTCTATACTTG TCAGGCCTCATCCTTCTCTTCTCTC	66

**Rt1**	GAGAGAAGAGAAGGATGAGCAGCACCCATGTCCTTAGT	38
**D1.1**	TGACGACAGCGATCTGTTGCCTGACTATCTTCTCACTCTATACTTGTCAGGCCTCATCCTTCTCTTCTCTC	71
**D1.2**	TGACGACAGCGATCTGTTGCCTGACCTCTATACTTGTCAGGCCTCATCCTTCTCTTCTCTC	61
**D1.3**	TGACGACAGCGATCTGTTGCCTGACTACTTGTCAGGCCTCATCCTTCTCTTCTCTC	56
**D1.4**	TGACGACAGCGATCTGTTGCTGACTCTCACTCTATACTTGTCAGCCTCATCCTTCTCTTCTCTC	64
**D1.5**	TGACGACAGCGATCTGTTGCACTGACTCTCACTCTATACTTGTCAGTGCCTCATCCTTCTCTTCTCTC	68
**D1.6**	TGACGACAGCGATTTGCCTGACTCTCACTCTATACTTGTCAGGCCTCATCCTTCTCTTCTCTC	63
**Rt1.6**	GAGAGAAGAGAAGGATGAGCACCCATGTCCTTAGT	35
**D1.7**	TGACGACAGCGATGTTGCCTGACTCTCACTCTATACTTGTCAGGCCTCATCCTTCTCTTCTCTC	64
**Rt1.7**	GAGAGAAGAGAAGGATGAGCCACCCATGTCCTTAGT	36
**D1.8**	TGACGACAGCGATCGTTGCCTGACTCTCACTCTATACTTGTCAGGCCTCATCCTTCTCTTCTCTC	65
**Rt1.8**	GAGAGAAGAGAAGGATGAGCGCACCCATGTCCTTAGT	37
**D1.9**	TGACGACAGCGATCATGTTGCCTGACTCTCACTCTATACTTGTCAGGCCTCATCCTTCTCTTCTCTC	67
**Rt1.9**	GAGAGAAGAGAAGGATGAGCATGCACCCATGTCCTTAGT	39
**T1**	GCTCGGAGACGACAAGTATAGAGTGAGAGTCAGGC	35
**FT1**	GCCTGACTCTCACTCTATACTTGTCGTCTCCGAGC	35
**D2**	CTGCAACAGCGATCTGTTGCCTGACAAGTATAGAGTGAGAGTCAGGCCTCATCCTTCTCTTCTCTC	66
**Rt2**	GAGAGAAGAGAAGGATGAGCAGCACCCATGTACACCTG	38
**T2**	GTACGATGCAGACTCTCACTCTATACTTGTCAGGC	35

**FT2**	GCCTGACAAGTATAGAGTGAGAGTCTGCATCGTAC	35
**RNA1**	ACTAAGGT/rA/GGTCGTCA	17
**RNA2**	CAGGTGTT/rA/GGTTGCAG	17
**RNA1**	ACTAAGGT/rA/GGTCGTCATTTTTTTTTTTTTTTTTT	35
**Q1**	GGTCGTCATTTTTTTTTTTTTTTTTT	26
